# Allosteric Conformational Locking of Sestrin2 by Leucine: An Integrated Computational Analysis of Branched-Chain Amino Acid Recognition and Specificity

**DOI:** 10.3390/molecules30244791

**Published:** 2025-12-16

**Authors:** Muhammad Ammar Zahid, Abbas Khan, Mona A. Sawali, Osama Aboubakr Mohamed, Ahmed Mohammad Gharaibeh, Abdelali Agouni

**Affiliations:** Department of Pharmaceutical Sciences, College of Pharmacy, QU Health, Qatar University, Doha P.O. Box 2713, Qatar; mz1912625@qu.edu.qa (M.A.Z.); abbas.khan@qu.edu.qa (A.K.); ms1800998@qu.edu.qa (M.A.S.); oa2003649@qu.edu.qa (O.A.M.); ag2107297@qu.edu.qa (A.M.G.)

**Keywords:** Sestrin2 (SESN2), leucine sensing, mTORC1 regulation, molecular dynamics simulations, allosteric stabilization

## Abstract

Sestrin2 (SESN2) is a highly conserved stress-inducible protein that serves as a central hub for integrating cellular responses to nutrient availability, oxidative stress, and endoplasmic reticulum (ER) stress. A key function of SESN2 is its role as a direct sensor for the branched-chain amino acid (BCAA) leucine, which modulates the activity of the mechanistic target of rapamycin complex 1 (mTORC1), a master regulator of cell growth and metabolism. While the functional link between leucine and SESN2 is well-established, the precise molecular determinants that confer its high specificity for leucine over other BCAAs, such as isoleucine and valine, remain poorly understood. This study employs an integrated computational approach, spanning atomic interactions to global protein dynamics, combining molecular docking, extensive all-atom molecular dynamics (MD) simulations, and binding free energy calculations, to elucidate the structural and dynamic basis of BCAA-SESN2 recognition. Our thermodynamic analysis reveals a distinct binding affinity hierarchy (Leucine > Isoleucine > Valine), which is primarily driven by superior van der Waals interactions and the shape complementarity of leucine’s isobutyl side chain within the protein’s hydrophobic pocket. Critically, a quantitative analysis of the conformational ensemble reveals that leucine induces a dramatic collapse of the protein’s structural heterogeneity. This “conformational locking” mechanism funnels the flexible, high-entropy unbound protein—which samples 35 distinct conformations—into a sharply restricted ensemble of just 9 stable states. This four-fold reduction in conformational freedom is accompanied by a kinetic trapping effect, which significantly lowers the rate of transitions between states. This process of conformational selection stabilizes a well-defined, signaling-competent structure, providing a comprehensive, atom-to-global-scale model of SESN2’s function. In the context of these findings, this work provides a critical framework for understanding SESN2’s complex role in disease and offers a clear rationale for the design of next-generation allosteric therapeutics.

## 1. Introduction

Branched-chain amino acids (BCAAs)—leucine, isoleucine, and valine—are essential nutrients that also act as potent signaling molecules regulating cell growth, metabolism, and homeostasis. However, this role is a double-edged sword; while vital for health, elevated BCAA levels are strongly linked to metabolic diseases, including obesity, insulin resistance, and type 2 diabetes. Central to this signaling network is Sestrin2 (SESN2), a highly conserved stress-inducible protein that functions as the primary cellular sensor for leucine. In the absence of leucine, SESN2 binds to and inhibits the GAP activity toward Rags 2 (GATOR2) complex, which in turn activates GAP activity toward Rags 1 (GATOR1) to suppress the mechanistic target of rapamycin complex 1 (mTORC1), a master regulator of anabolic processes [1,2,3]. Upon binding leucine with high affinity, SESN2 undergoes a conformational change, releases GATOR2, and unleashes mTORC1 activity. This mechanism establishes SESN2 as a critical metabolic checkpoint, but its remarkable specificity for leucine over other structurally similar BCAAs remains poorly understood at a molecular level.

Significant structural and mechanistic questions persist, forming a critical gap in our understanding. While the crystal structure of leucine-bound SESN2 has been solved, repeated attempts to crystallize the protein in its unbound (apo) state have failed, strongly suggesting that the apo-protein is intrinsically flexible and exists in a conformationally heterogeneous state [4,5,6]. Furthermore, recent cryogenic electron microscopy (cryo-EM) has revealed the massive, cage-like architecture of the GATOR2 complex and identified its interaction site with SESN2. Yet, the fundamental allosteric question remains unanswered: how does the binding of a small leucine molecule within a buried pocket in SESN2 trigger a large-scale structural change that causes its dissociation from the vast GATOR2 complex? This knowledge gap highlights the need to elucidate the dynamic, allosteric communication that links the ligand-binding event to global protein function.

The urgency to fill this gap is underscored by SESN2’s multifaceted roles and recent clinical setbacks. Beyond mTORC1, SESN2 is a pleiotropic regulator of cellular health, activating the Nrf2 antioxidant response and mitigating endoplasmic reticulum (ER) stress through mTORC1-independent pathways. This complexity likely contributed to the recent clinical trial failure of NV-5138, a SESN2-targeting drug designed to activate mTORC1 for treating depression [7,8,9]. The disappointing outcome suggests that a simple “on/off switch” model of SESN2 is insufficient and that therapeutic success may depend on recapitulating the specific conformational state induced by its natural ligand, leucine, which orchestrates a balanced response across all its downstream pathways. A foundational, atomic-level model of ligand recognition and allostery is therefore urgently needed.

Here, we hypothesize that the specificity of SESN2 for leucine is not merely a result of static contacts in the binding pocket, but is dictated by a unique dynamic process of “conformational locking.” We propose that only leucine possesses the precise shape complementarity required to overcome the entropic penalty of stabilizing the flexible apo-protein, locking it into a singular, low-energy, and signaling-competent state. To test this hypothesis, this study employs an integrated computational approach that combines molecular docking, extensive all-atom molecular dynamics (MD) simulations, and binding free energy calculations. Our objective is to provide a detailed, atomic-to-global scale dissection of the structural, energetic, and dynamic basis for SESN2’s function as a highly specific leucine sensor, thereby creating a framework for understanding its complex role in health and for the rational design of next-generation therapeutics.

## 2. Results and Discussion

### 2.1. Generation and Validation of a Full-Length SESN2 Model

Construction of a full-length SESN2 model was essential for studying conformational dynamics involving unresolved regions missing from the 5DJ4 crystal structure. Integration of the AlphaFold-predicted loops into the crystallographic template produced a complete structure that preserved the SESN2 folds while reinstating key flexible regions. Structural alignment of the final minimized hybrid model with the original 5DJ4 coordinates demonstrated excellent conservation of the global architecture, particularly within the C-terminal domain (residues 339–480), which encloses the leucine-binding site. This domain exhibited a backbone root-mean square deviation (RMSD) of only 0.88 Å, indicating that the incorporation and subsequent minimization of the reconstructed segments did not perturb the core structure. Although the N-terminal and internal linker segments displayed greater conformational variability, consistent with their intrinsic flexibility, their incorporation did not disrupt local packing arrangements. These observations confirm that the hybrid structure retains the experimentally supported three-dimensional framework while providing a physically realistic representation of previously unresolved regions. The final model, therefore, provides a robust starting point for molecular docking and MD simulations to characterize the dynamic roles of these flexible segments in SESN2 function.

### 2.2. Molecular Docking Validates Binding Pose and Informs Initial BCAA Interactions

**Figure 1:**
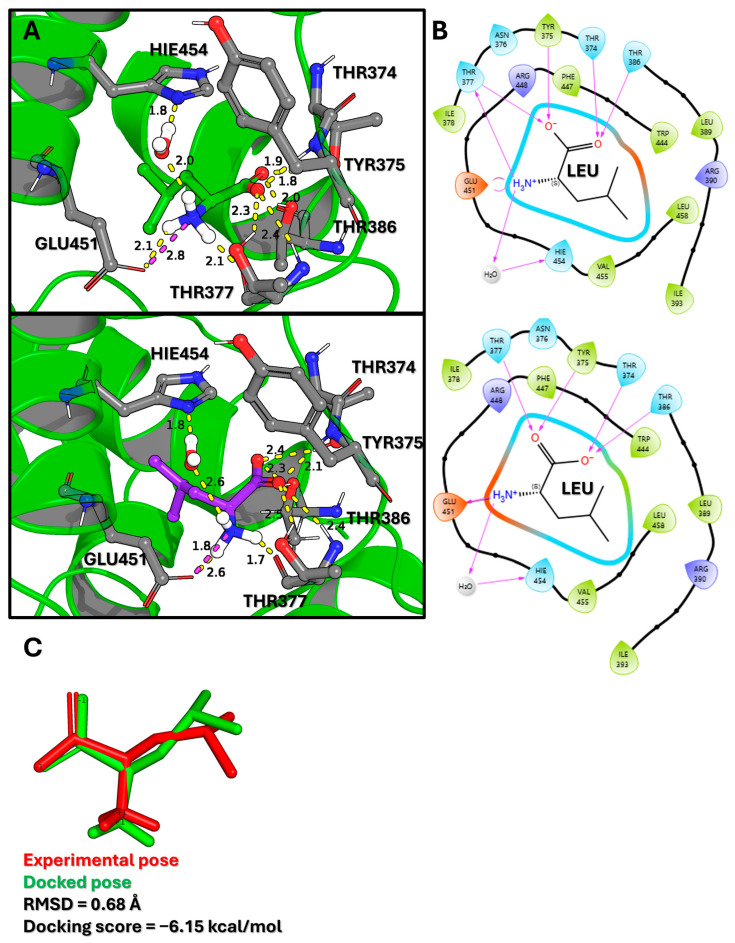
Validation of the Leucine Binding Mode through Molecular Docking. (**A**) 3D visualization comparing the computationally docked pose of leucine (top) with its experimentally determined crystallographic pose (bottom). Green ribbons denote the SESN2 protein backbone, and grey sticks represent carbon atoms of key interacting residues. Yellow dashed lines indicate hydrogen bonds, with adjacent numbers specifying the interaction distances in Angstroms (Å). (**B**) Corresponding 2D ligand interaction diagrams for the docked (top) and experimental (bottom) poses. Residue spheres are colored by chemical property: green (hydrophobic), blue (polar), purple (basic), and orange (acidic). Purple arrows indicate hydrogen bond directionality (donor to acceptor). (**C**) Structural alignment of the experimental (red) and docked (green) leucine poses. The low root-mean-square deviation (RMSD) of 0.68 Å confirms the high accuracy of the docking protocol, while the docking score of −6.15 kcal/mol indicates a favorable theoretical binding affinity.

Molecular docking was performed to generate initial energetically favorable binding poses and to validate the computational setup. The protocol was first validated by re-docking the native ligand, leucine, into the SESN2 binding site. As shown in Figure 1, the resulting docked pose showed excellent agreement with the experimentally determined crystallographic pose, yielding an RMSD of only 0.68 Å. This confirms that the docking protocol can reliably reproduce the correct binding orientation. Analysis of the docked pose revealed extensive hydrogen bonding and polar contacts with key residues, including THR374, TYR375, THR377, GLU451, and HIS454, all of which were conserved in the experimental structure.

**Figure 2:**
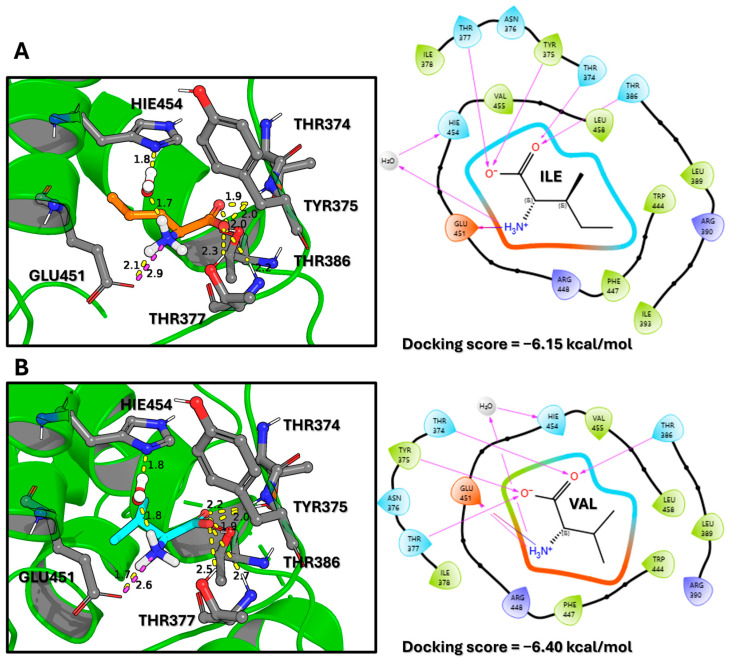
Predicted Binding Modes and Interaction Profiles of Isoleucine and Valine. (**A**) 3D visualization of the docked isoleucine ligand (orange sticks) within the SESN2 binding pocket (green ribbons). Interacting amino acid residues are shown as grey sticks. Yellow dashed lines represent hydrogen bonds, with adjacent numbers indicating bond lengths in Angstroms (Å). The corresponding 2D interaction diagram (right) depicts residue spheres colored by chemical property: green (hydrophobic), blue (polar), purple (basic), and orange (acidic). Purple arrows denote hydrogen bond directionality (donor to acceptor). (**B**) 3D visualization of the docked valine ligand (cyan sticks) and its 2D interaction diagram (right), which follows the same color scheme as in (**A**). Both ligands maintain a conserved interaction network anchored by residues GLU451 and HIS454. The favorable docking scores (−6.15 kcal/mol for isoleucine and −6.40 kcal/mol for valine) suggest strong binding affinities, comparable to that of leucine.

Following this validation, the same protocol was used to predict the binding modes of isoleucine and valine (Figure 2). The docking scores for leucine, isoleucine, and valine were −6.15 kcal/mol, −6.15 kcal/mol, and −6.40 kcal/mol, respectively. These scores suggest comparable binding affinities, with valine even appearing slightly more favorable. This highlights a known limitation of docking scoring functions: while excellent for pose prediction, they may lack sufficient sensitivity to rank the binding affinities of closely related ligands accurately. Nevertheless, the docking successfully identified a conserved binding mode for all three BCAAs, anchored by interactions with GLU451 and HIS454, with their side chains occupying the hydrophobic pocket. These validated poses served as ideal initial configurations for the extensive all-atom MD simulations required to dissect the subtle energetic and dynamic differences that govern specificity.

### 2.3. MD Simulations Reached Stable Equilibrium and Were Reproducible

**Figure 3:**
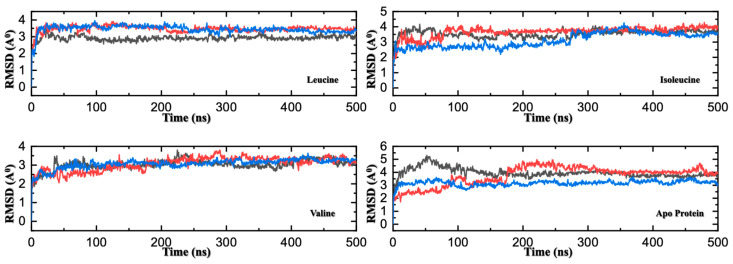
RMSD Comparison of Three MD Replicates for Apo and Ligand-Bound SESN2. Root-mean-square deviation (RMSD) trajectories of backbone atoms for three independent 500 ns molecular dynamics replicates of SESN2 in its apo form and in complex with leucine, isoleucine, or valine. Each panel corresponds to one system, with three colored traces (red, blue, and black) representing individual replicates. All systems reach equilibration within ~25 ns. Leucine- and valine-bound systems display low RMSD variability across replicates, indicating high structural stability and reproducibility. In contrast, the apo and isoleucine-bound forms exhibit greater fluctuations and greater divergence between replicates, reflecting increased conformational flexibility and reduced stabilization upon binding.

Before dissecting the specific molecular interactions, we first validated the convergence and reproducibility of the extensive MD simulations that underpin this study. For each of the four systems (apo, leucine-bound, isoleucine-bound, and valine-bound), three independent 500 ns simulations were performed to ensure robust sampling. The stability of each system was assessed by monitoring key structural and energetic parameters over time. The RMSD of the protein Cα atoms for all systems stabilized after an initial relaxation period (typically within ~50 ns), indicating that the simulations reached a stable structural equilibrium (Figure 3). Furthermore, the total and potential energies of each system remained stable throughout the simulation runs, confirming thermodynamic equilibration.

**Figure 4:**
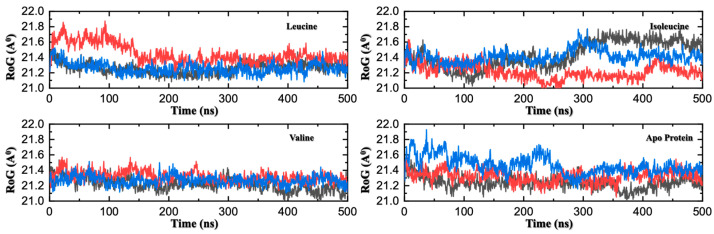
Radius of Gyration (Rg) Comparison Across Three MD Replicates of Apo and Ligand-Bound SESN2. Time evolution of the Rg for three independent 500 ns MD replicates. Each panel corresponds to one system, with three colored traces (red, blue and black) representing individual replicates. While the average Rg is similar across all systems, the leucine- and valine-bound complexes exhibit markedly reduced fluctuations and more stable Rg values over time. This demonstrates that ligand binding locks the protein into a conformationally stable state, dampening the large-scale motions observed in the more flexible apo and isoleucine-bound forms.

Crucially, the trajectories from the three independent replicates for each system spanned a narrow, consistent range of conformational space, as reflected by low variance in both RMSD and Radius of Gyration (Rg) (Figure 3 and Figure 4). This high degree of consistency demonstrates the reproducibility of our results and provides confidence that the observed conformational states are representative. The trajectories from all three independent replicates for each system are presented directly in the subsequent figures to demonstrate the reproducibility of our results and provide a clear view of the conformational sampling.

### 2.4. Thermodynamic Analysis Reveals Leucine as the Preferential Ligand for SESN2

A quantitative basis for the binding specificity of BCAAs to SESN2 was established by first determining their relative binding affinities. We employed the Molecular Mechanics/Generalized Born Surface Area (MM/GBSA) method, a robust computational technique for estimating the binding free energy (ΔG_bind_) from MD simulation trajectories. A more negative ΔG_bind_ value indicates a more stable, thermodynamically favorable interaction. The calculations were performed on the final, equilibrated portion of three independent 500 ns simulations for each complex, ensuring the results are statistically sound.

The computed binding free energies, presented in Table 1, reveal a clear and distinct hierarchy of binding affinity among the three BCAAs. Leucine binds to SESN2 with the highest affinity, exhibiting a ΔG_bind_ of −37.60 ± 2.39 kcal/mol. This is followed by isoleucine, with a moderately strong affinity of −34.47 ± 1.98 kcal/mol. Valine demonstrates the weakest interaction, with a ΔG_bind_ of −30.32 ± 3.18 kcal/mol. Acknowledging the standard deviations observed across the three replicates, we performed independent two-sample *t*-tests to assess whether the differences in binding free energies were statistically significant. The analysis revealed a ~7.3 kcal/mol advantage of leucine over valine (*p* = 0.034), providing strong quantitative support for SESN2’s specificity. This substantial thermodynamic advantage is the primary driver of specificity, ensuring that, under physiological conditions, leucine preferentially occupies the binding site over other BCAAs to initiate the signaling cascade.

In contrast, the minor difference between leucine and isoleucine (~3.1 kcal/mol) did not reach statistical significance (*p* = 0.155) with the current sample size of three replicates. This suggests that while a clear binding preference for leucine over isoleucine is observed, a larger number of simulations would be required to establish this difference with high statistical confidence. Nevertheless, the overall computationally derived binding order—with leucine as the most potent binder—is in excellent agreement with experimental evidence that firmly establishes SESN2 as the primary cellular sensor for leucine [10].

The energetic contributions that govern these differential affinities were dissected by decomposing the total binding free energy into its constituent energetic terms (Table 1). This analysis provides insight into the nature of the BCAA-SESN2 interaction. The most significant favorable contribution to binding for all three ligands comes from van der Waals energy (ΔEvdW), which reflects non-polar interactions such as dispersion forces and serves as a proxy for shape complementarity. The magnitude of this term directly correlates with the binding affinity hierarchy: leucine shows the most favorable ΔEvdW (−28.09 ± 1.28 kcal/mol), followed by isoleucine (−25.92 ± 1.38 kcal/mol), and valine (−21.96 ± 1.29 kcal/mol). This trend strongly suggests that the primary determinant of binding strength is the ability of the BCAA’s aliphatic side chain to maximize favorable contacts within the hydrophobic binding pocket of SESN2. Leucine’s larger isobutyl side chain provides the greatest surface-area contact, leading to the strongest interaction.

Conversely, the electrostatic contribution, represented by the Coulomb energy (ΔE_Coulomb_), is unfavorable for all three complexes. This indicates that the binding process likely involves a significant desolvation penalty for the charged amino and carboxyl groups of the BCAAs as they move from the aqueous solvent into the more confined binding pocket. While these groups facilitate favorable interactions within the pocket, they are insufficient to offset the energy cost of removing their hydration shells. The solvation energy terms, particularly the favorable lipophilic energy (ΔG_Lipo_), further reinforce the dominance of hydrophobicity in driving the binding event. Finally, the calculated ligand strain energy was minimal for all three BCAAs, indicating that they can adopt their bound conformation without incurring a significant energetic penalty. In summary, the thermodynamic analysis establishes that SESN2 preferentially binds leucine due to stronger hydrophobic and van der Waals interactions, providing the quantitative foundation for a structural and dynamic explanation of this specificity.

### 2.5. Leucine Binding Induces Allosteric Stabilization of SESN2

Having established that leucine binds to SESN2 with the highest affinity, the next important question is to understand the structural consequences of this binding event. To investigate this, we analyzed the overall structural stability of SESN2 in its unbound (apo) state and in complexes with each of the three BCAAs, using RMSD and Rg as metrics. The RMSD of the protein’s Cα atoms measures the deviation of the backbone from its initial structure, providing a quantitative assessment of conformational stability over time. The Rg measures the protein’s overall size and shape, indicating whether it undergoes global conformational change [11]. The convergence of the trajectories in independent MD simulation runs was confirmed before proceeding to further analysis. The RMSD analysis in Figure 3 shows ligand-induced stabilization. The apo-SESN2 protein consistently exhibits the highest RMSD values across the simulations, with large fluctuations centered on a mean of ~3.5–4.0 Å. This indicates that the unbound protein is intrinsically flexible and dynamically samples a broad range of conformations, which is consistent with the experimental difficulty of obtaining a crystal structure of the apo state [4]. In contrast, binding any of the three BCAAs reduces RMSD, indicating that ligand engagement stabilizes the protein and restricts its conformational freedom.

A clear hierarchy of stabilization emerges, mirroring the thermodynamic affinities determined by MM/GBSA. The leucine-bound complex is the most stable, settling into a low and very stable RMSD plateau around ~2.5–3.0 Å. The valine-bound complex is also highly stable, with a similar RMSD profile, whereas the isoleucine-bound complex is intermediate in stability, with a slightly higher, more fluctuating RMSD. This robust correlation between binding energy and structural rigidity suggests that the more tightly a ligand binds, the more effectively it dampens the protein’s intrinsic motions and locks it into a stable fold.

The Rg analysis reveals a key consequence of ligand binding: a significant stabilization of the protein’s overall structure, as shown in Figure 4. The apo-protein exhibits substantial fluctuations in its Rg, with an average of ~21.4–21.5 Å, indicating that it exists in a dynamic, conformationally flexible state. Upon binding leucine or valine, these fluctuations are markedly dampened, and the protein settles into a more stable state with a consistent Rg of ~21.3 Å. While the change in the average size is subtle, the reduction in dynamic variability is significant. This effect is characteristic of a ‘conformational selection’ mechanism, where the ligand does not induce a large-scale compaction but rather stabilizes a pre-existing, signaling-competent conformation from the protein’s dynamic ensemble. This stabilization is the hallmark of an allosteric mechanism, providing a plausible explanation for how a local binding event can remodel and stabilize distant functional interfaces, such as the one required for GATOR2 interaction. Notably, this stabilizing effect is less pronounced for isoleucine, whose Rg profile remains more flexible and similar to the apo state, providing a structural rationale for SESN2’s ligand specificity.

### 2.6. A Dynamic Allosteric Network Communicates the Ligand Binding Event Across the Protein

The observed conformational change upon binding of leucine and valine raises the question of how this signal is propagated throughout the protein structure. To map these pathways of intramolecular communication, we analyzed residue-level flexibility and correlated motions within the protein using root-mean-square fluctuations (RMSF) and a dynamic cross-correlation matrix (DCCM).

**Figure 5:**
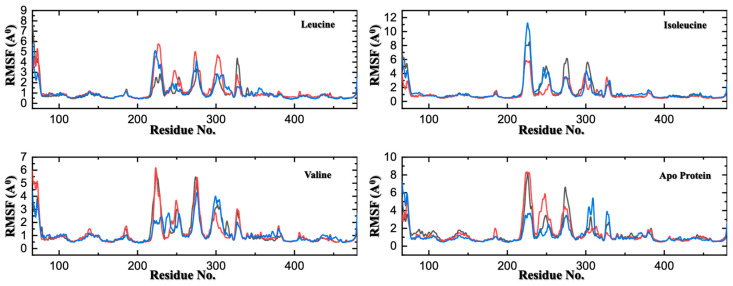
Residue-Level Flexibility Analysis via Cα-RMSF Across Three MD Replicates. Root-mean-square fluctuation (RMSF) profiles of Cα atoms, with each colored line (red, blue, and black) in the panels representing one of the three independent 500 ns simulations for SESN2 in its apo form and in complex with leucine, isoleucine, or valine. The analysis shows consistent flexibility patterns across the replicates. Leucine and valine binding not only rigidifies residues in the binding pocket but also allosterically dampens the flexibility of distant loop regions (e.g., residues ~240–260). In contrast, the apo and isoleucine-bound forms exhibit significantly greater mobility in these regions, indicating weaker conformational restraint.

The RMSF analysis, which measures the fluctuation of each residue, provides a high-resolution map of protein dynamics. As expected, binding of all three BCAAs dramatically reduces the flexibility of residues within the binding pocket, physically constraining their motion (Figure 5). However, the most significant insight comes from examining regions distant from the binding site. The apo-protein displays several prominent peaks of high flexibility, corresponding to mobile loop regions that connect the structural domains of SESN2 (e.g., residues ~240–260 and ~320–340). Upon leucine binding, the fluctuations in these distant loops are markedly dampened. This reduction in flexibility in regions far removed from the ligand provides direct, compelling evidence of an allosteric communication network. The binding event in the Sesn-C domain does not remain localized; instead, it transmits a stabilizing signal that propagates throughout the protein’s architecture, rigidifying its overall structure. This allosteric stabilization is the likely mechanism by which leucine binding remodels the conformation of the GATOR2 binding interface, which is known to be spatially distinct from the leucine pocket, to cause its dissociation.

**Figure 6:**
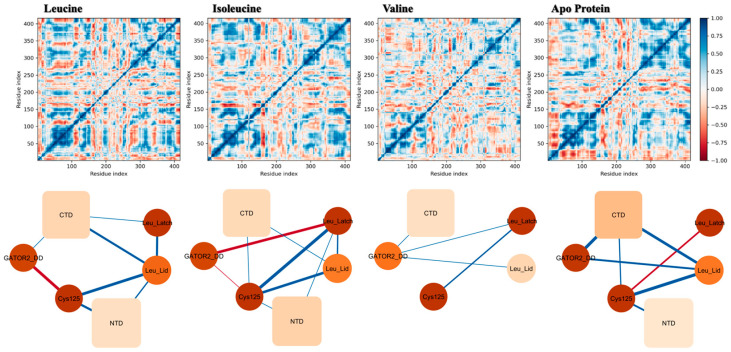
Dynamic Cross-Correlation Analysis Reveals a Leucine-Specific Allosteric Communication Network. (Top Panels) Dynamic cross-correlation matrices (CCMs) showing pairwise residue motion correlation computed from concatenated MD trajectories. Strong positive correlations (coordinated motion) are colored deep blue, whereas strong negative correlations (anti-correlated motion) are deep red. (Bottom Panels) Simplified network models representing the average correlations between key functional domains. Nodes represent the C-terminal domain (CTD), N-terminal domain (NTD), the GATOR2 docking domain (GATOR2_DD), the redox-sensitive cysteine (Cys125), and leucine-binding microdomains (Leu_Lid, Leu_Latch). The thickness of the edges is proportional to the correlation strength (blue for positive, red for negative). The analysis reveals that only leucine binding establishes a strong, fully connected allosteric network, providing a physical pathway for the binding signal to be transmitted to distal functional sites.

To map the pathways of intramolecular communication and understand how the ligand-binding signal is propagated, we analyzed correlated motions using DCCM analysis. To distill complex residue-level data into a clear picture of allosteric regulation, we constructed simplified network models that capture the average correlations among key functional domains (Figure 6). This analysis reveals a clear ligand-dependent activation gradient (Leucine > Isoleucine >> Valine ≈ Apo), where only leucine optimally organizes the protein’s dynamic network for full allosteric communication.

The unbound apo-protein (Figure 6, far right) exhibits a baseline level of internal communication, reflecting its native flexibility. However, its global network is suboptimal, characterized by diffuse correlations and a lack of organized long-range communication. Similarly, valine binding provides minimal stabilization, resulting in a fragmented, sparsely connected network in which the leucine-binding microdomains and the CTD are largely disconnected from other functional regions.

In contrast, isoleucine binding elicits a partial activation, maintaining some core network connections (e.g., between Cys125 and the CTD) but failing to propagate dynamic communication as efficiently as leucine, particularly to the NTD and the GATOR2 docking domain.

The binding of leucine (Figure 6, far left) induces the highest degree of coordinated dynamics, transforming the protein into a fully activated allosteric network. The DCCM shows the strongest and most structured positive correlations, with extensive long-range communication. The network model reveals that leucine establishes a strong allosteric axis centered around the Cys125, CTD, Leu_Lid, and GATOR2-interacting domains. This dense web of communication provides a physical pathway for the binding signal to be transmitted to distal functional sites. These results highlight that, although SESN2 possesses an intrinsic capacity for dynamic coupling, binding only its primary ligand, leucine, can fully organize and stabilize the specific allosteric network required for maximal biological activity.

Taken together, these analyses reveal that leucine binding does not merely stabilize SESN2 but actively organizes its internal dynamics into a coherent, allosterically coupled network. This network provides the physical means to transmit the signal of leucine’s presence from the sensory pocket to distal functional sites. This allosteric communication is fundamental to understanding SESN2’s role as a multi-functional signaling hub. The same network that likely modulates the GATOR2 interface may also interact with other functional regions, such as the Sesn-A domain, which contains the Cys125 residue critical for its antioxidant activity [12]. This provides a structural framework for hypothesizing how the binding of a single amino acid could simultaneously and differentially regulate SESN2’s distinct roles in metabolic signaling and redox homeostasis.

### 2.7. Atomic Interactions in the Binding Pocket Dictate Ligand Specificity and Stability

**Figure 7:**
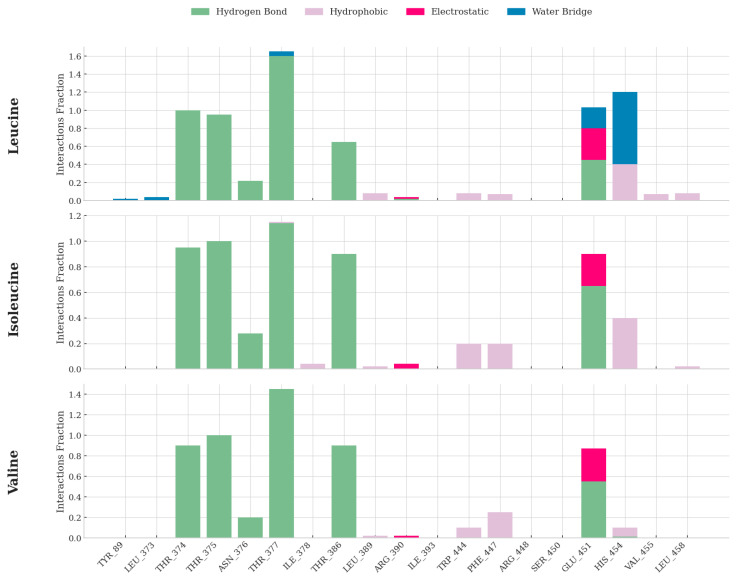
Quantitative Analysis of Protein–Ligand Interactions Throughout MD Simulations. Interaction fraction plots showing the percentage of simulation time that specific interactions—hydrogen bonds (green), hydrophobic contacts (purple), electrostatic interactions (red), and water bridges (blue)—are maintained between SESN2 residues and each bound ligand. The data demonstrates that leucine forms the most extensive, diverse, and persistent set of interactions, providing an atomic-level explanation for its superior binding affinity.

We performed a detailed analysis of the atomic-level interactions within the binding pocket and assessed the stability of the ligands themselves to uncover the ultimate origin of SESN2’s binding specificity. This provides the foundational, chemical explanation for the thermodynamic and dynamic differences observed among the three BCAAs. Our analysis of the protein–ligand interaction networks across the simulations reveals a set of conserved contacts that anchor the common backbone of all three amino acids. The charged amino and carboxyl groups of the BCAAs are consistently stabilized by a network of hydrogen bonds and water-mediated bridges, primarily involving the side chains of residues Glu451 and His454, and a cluster of threonine residues (THR374, THR375, THR377) (Figure 7). This common anchoring motif ensures that each BCAA is appropriately positioned within the binding cleft, presenting its unique side chain for recognition. The critical determinants of specificity and affinity, however, lie in the differential interactions between the hydrophobic side chains and the pocket’s nonpolar lining. The nature and extent of these contacts vary significantly for each amino acid. Leucine forms the most diverse and robust interaction network, which accounts for its superior binding affinity. Its binding is characterized by multiple persistent hydrogen bonds with a cluster of threonine residues (THR374, THR375, THR377) and ASN376. This polar network is complemented by crucial water bridges and electrostatic interactions with HIS454 and GLU451, which further anchor the ligand. Finally, its isobutyl side chain forms extensive hydrophobic contacts with residues like VAL453 and, critically, ILE378. This dense, multivalent network of polar, hydrophobic, and water-mediated interactions provides a powerful enthalpic driving force for binding. Isoleucine retains some key interactions, including hydrogen bonds with the threonine cluster and contacts with GLU451 and HIS454. However, its interaction profile is less cooperative. It forms fewer stabilizing water bridges, and its sec-butyl side chain makes fewer hydrophobic contacts than leucine. This results in a moderate, but significantly weaker, interaction network. Valine’s smaller isopropyl side chain severely restricts its interaction footprint. While it maintains strong hydrogen bonding with the threonine cluster, it forms significantly fewer interactions with HIS454 and with key hydrophobic residues, such as ILE378, VAL455, and LEU458. This results in a more straightforward, less robust binding interface, as evidenced by the lowest binding free energy.

**Figure 8:**
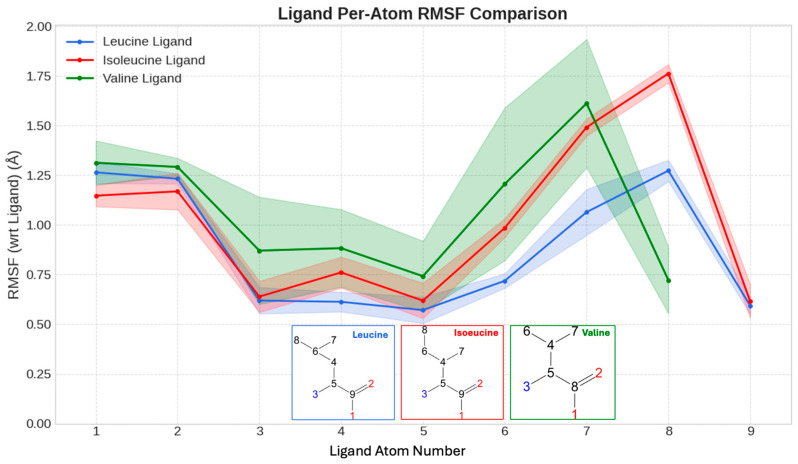
Positional Stability of Bound Ligands within the SESN2 Pocket. Per-atom root-mean-square fluctuation (RMSF, in Å) for the heavy atoms of leucine, isoleucine, and valine during their respective simulations. Lower RMSF values indicate a more rigidly held, stable atom. Inset diagrams show the atom numbering used for the RMSF calculation on the x-axis. Leucine’s atoms exhibit the lowest fluctuations, confirming that its extensive interaction network tightly anchors it. The data reveal a clear ligand-stability hierarchy (Leucine < Isoleucine < Valine) that mirrors the binding affinities.

This narrative of differential fit is in accordance with the analysis of the per-atom RMSF of the bound ligands themselves. This metric quantifies the positional stability of each atom within the ligand, where lower RMSF values indicate a more rigidly held atom. The backbone atoms exhibit relatively low, comparable fluctuations across all three ligands, confirming stable anchoring, whereas the key distinction, once again, lies in the side chains (Figure 8). Leucine exhibits the lowest overall RMSF, with its central backbone atoms being exceptionally stable (<0.6 Å), confirming that its extensive interaction network tightly anchors it. In contrast, valine shows the highest average RMSF, with its side chain atoms fluctuating up to ~1.9 Å, a direct consequence of its sparse contacts and weaker anchoring. Isoleucine displays intermediate flexibility, with its terminal side chain being notably less restrained than leucine’s. This hierarchy of ligand stability (Leucine < Isoleucine < Valine) is a direct physical manifestation of the quality of their respective interaction networks. It provides a compelling, atomic-level explanation for the observed binding affinities and the differential ability of these ligands to stabilize the entire protein structure.

### 2.8. Ligand Binding Drives a Quantitative Collapse of the Conformational Landscape

To synthesize our structural, dynamic, and energetic findings into a unified thermodynamic picture, we analyzed the conformational ensembles using PCA and density-based clustering. This approach enabled a direct, quantitative comparison of the energy landscapes of the apo-protein and ligand-bound forms.

**Figure 9:**
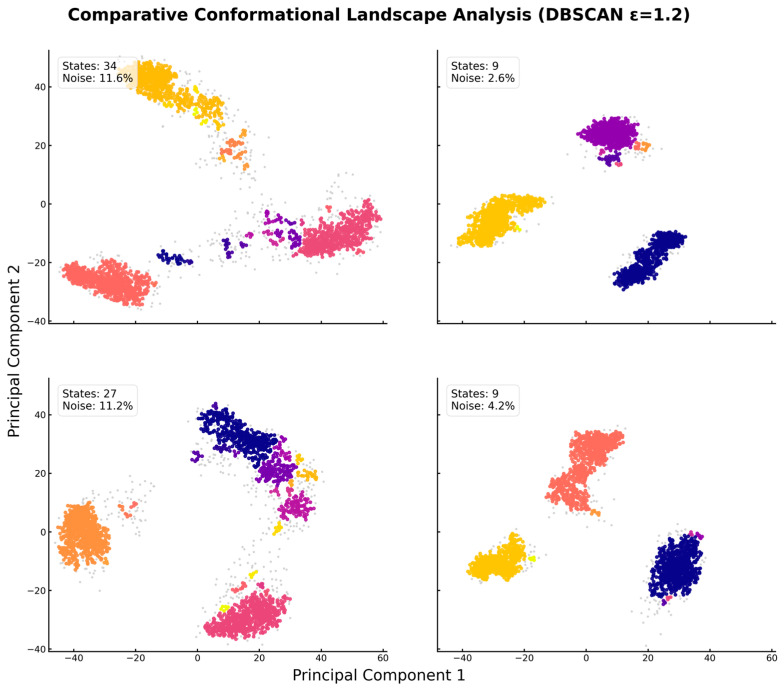
Quantitative Clustering of Conformational Landscapes via DBSCAN Analysis Reveals Ligand-Induced Collapse. Projection of the Cα atom trajectories onto the first two principal components (PC1 and PC2), with distinct colors in each panel representing unique stable conformational states identified by the clustering algorithm, and gray points representing transitional noise. (Top-Left) The apo-protein explores a vast landscape, sampling 34 distinct states with significant time (11.6%) in transitional conformations. (Top-Right) Leucine binding induces a dramatic collapse, restricting the protein to just 9 well-defined states and minimizing time spent in transitions (2.6% noise). (Bottom-Left) The isoleucine-bound form remains conformationally heterogeneous, similar to the apo state (27 states). (Bottom-Right) Valine binding also restricts the landscape to 9 stable states. In each panel, distinct colors represent unique stable conformational clusters, while gray points represent transitional “noise” conformations that do not belong to any stable state. This analysis provides direct, quantitative evidence of the “conformational locking” mechanism driven by leucine and valine.

Our analysis reveals profound, ligand-specific differences in conformational heterogeneity, as summarized in Table 2 and visualized in Figure 9. The unbound apo-protein explores a vast, rugged energy landscape, sampling 35 distinct conformational states. This is characteristic of a flexible, high-entropy ensemble, consistent with its high RMSD and the experimental difficulty in obtaining an apo-state crystal structure. The isoleucine-bound form remains similarly heterogeneous, occupying 27 distinct states, indicating that its binding is insufficient to restrict the protein’s intrinsic flexibility substantially.

In contrast, the binding of leucine or valine induces a dramatic collapse of the conformational landscape. Both ligands reduce the number of accessible stable states by approximately four-fold to just 9 (Table 2). This is visually represented in Figure 9, where the sprawling, scattered distribution of the apo and isoleucine systems is funneled into a few, tightly packed conformational islands for the leucine and valine complexes. This demonstrates that only specific ligands can drive the large-scale conformational selection required to stabilize the protein. However, this reduction in conformational states is not, by itself, sufficient for biological activity. The key differentiator lies in the quality of the resulting stabilized ensemble. As revealed by our dynamic network analysis (Figure 6), only leucine binding establishes a strong, fully connected allosteric communication network capable of propagating the binding signal to distal functional sites. While valine can stabilize the protein, its resulting allosteric network is fragmented and suboptimal, failing to organize the protein for downstream signaling efficiently. Furthermore, leucine is more effective at kinetically trapping the protein, reducing the time spent in transitional states to just 2.6% compared to 4.2% for valine (Table 2). Therefore, SESN2’s specificity for leucine arises not just from its ability to bind tightly, but from its unique capacity to lock the protein into a thermodynamically stable and allosterically competent signaling state.

Beyond reducing the number of states, leucine and valine also fundamentally alter the protein’s dynamics. The apo and isoleucine forms spend more than 11% of their simulation time in low-density “noise” regions that correspond to high-energy transition states between stable conformations. Upon leucine binding, this value plummets to a mere 2.6% (Table 2). This indicates that leucine not only reduces the static conformational entropy but also imposes a kinetic barrier, effectively trapping or “locking” the protein in its stable basins and preventing exploration of the broader landscape.

**Figure 10:**
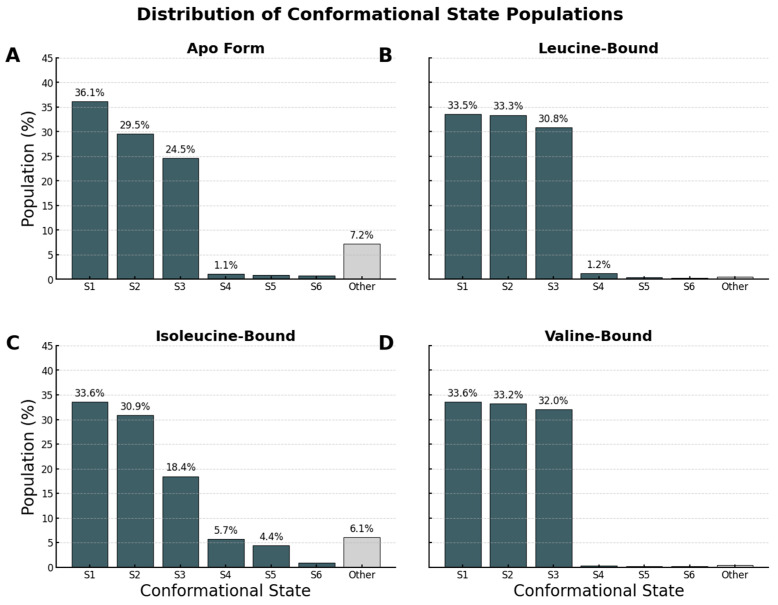
Quantitative Population Analysis of Conformational States. Distribution of conformational state populations identified by DBSCAN clustering of the trajectories projected onto PC1 and PC2. In all panels, dark grey bars represent distinct stable macrostates (S1–S6), while the light grey “Other” bar aggregates the remaining low-population microstates and transitional noise. (**A**) The Apo form exhibits a broad distribution, with a large “Other” population (7.2%), consistent with high entropy. (**B**) Leucine binding consolidates the ensemble into three dominant states (S1–S3). (**C**) The Isoleucine-bound system retains significant heterogeneity, similar to that of the apo form. (**D**) Valine binding mirrors the leucine effect, showing a stabilized landscape dominated by three major states. These data provide quantitative evidence that leucine and valine drive a conformational locking mechanism, whereas isoleucine fails to stabilize a specific, distinct conformation.

The distribution of state populations further underscores this principle of conformational funneling (Figure 10). The apo and isoleucine systems exhibit a characteristic long-tail distribution, with dozens of lowly populated microstates, the fingerprint of a high-entropy system. Conversely, the distributions for the leucine and valine systems are dominated by only three highly populated macrostates, which together account for more than 95% of the total population. This provides clear, quantitative evidence that specific ligand binding overcomes the protein’s intrinsic entropy to select for and stabilize a small, well-defined ensemble of functional conformations.

## 3. Materials and Methods

### 3.1. Protein Structure Modeling and Preparation

The experimentally determined X-ray crystal structure of the human SESN2 core domain bound to leucine (PDB ID: 5DJ4) served as the primary template for this study. Although 5DJ4 successfully captures the global fold and ligand-binding architecture, several flexible regions were unresolved, including the N-terminal 65 residues and three internal linker loops spanning residues 242–255, 272–280, and 296–309. These segments are likely to influence inter-domain communication, conformational flexibility, and potential allosteric behavior; therefore, a complete model was required to enable meaningful downstream simulations. A full-length hybrid structure was generated by reconstructing the missing segments using high-accuracy structural predictions from AlphaFold2 [13]. The predicted coordinates for the absent residues were grafted into the 5DJ4 template, while preserving the experimentally observed atomic positions in all resolved regions. Loop insertion was guided by sequence alignment and local structural context to minimize backbone discontinuities, ensuring that the composite model retained the experimental fold while restoring the disordered and flexible motifs essential for dynamic analyses. The resulting chimeric structure was prepared using the Protein Preparation Wizard in Maestro (version 14.5) within the Schrödinger Suite (Schrödinger Release 2025-1) [14]. Hydrogen atoms and proper bond orders were assigned, protonation states were set at physiological pH, disulfide linkages were verified, and local steric clashes at the graft junctions were corrected. A restrained energy minimization with the OPLS4 force field was then performed to relieve local strain and optimize the hydrogen-bonding network, particularly around the reconstructed regions [15]. To further refine the model, the minimized hybrid structure was subjected to a best-practice relaxation protocol in Desmond. The system first underwent vacuum relaxation via steepest-descent minimization, with heavy-atom restraints applied to the crystallographically defined core to relieve short-range steric clashes without distorting the native fold. The model was then solvated in a TIP3P water box with 10 Å padding and neutralized; a second minimization stage was performed with gradually reduced positional restraints to allow proper relaxation of solvent–loop interactions. Finally, the system was subjected to a short, restrained equilibration under NVT conditions, followed by NPT conditions, allowing the AlphaFold-derived regions to equilibrate while maintaining the original 5DJ4 structural framework.

### 3.2. Ligand Preparation

The three-dimensional structures of the branched-chain amino acids L-leucine, L-isoleucine, and L-valine were prepared using the LigPrep tool within Maestro (version 14.5), Schrödinger Suite (Schrödinger Release 2025-1: LigPrep, Schrödinger, LLC, New York, NY, USA, 2025). This step is crucial for generating accurate, low-energy 3D ligand conformations. The process included the generation of possible ionization states at a physiological pH of 7.4 ± 0.2 using Epik (version 7.3) (Schrödinger Release 2025-1: Epik, Schrödinger, LLC, New York, NY, USA, 2025), followed by the generation of tautomers and stereoisomers [16]. The geometry of each resulting ligand structure was then optimized using the OPLS4 force field to ensure it represented a stable, low-energy conformation suitable for docking and simulation studies [15].

### 3.3. Molecular Docking

To predict the initial binding poses of the BCAAs, a targeted molecular docking study was performed using the Glide (version 10.8) program in extra precision (XP) mode from the Schrödinger Suite (Schrödinger Release 2025-1: Glide, Schrödinger, LLC, New York, NY, USA, 2025) [17,18,19]. A receptor grid was generated by defining a bounding box centered on the coordinates of the co-crystallized leucine ligand within the known binding site of the 5DJ4 crystal structure. This targeted approach was selected over a blind docking study because the specific leucine-binding pocket is well established experimentally. The primary goal was not to discover novel binding sites, but rather to accurately generate initial binding poses for isoleucine and valine within this known active site and to validate the protocol by re-docking leucine, thereby creating reliable starting structures for the subsequent MD simulations. The docking protocol employed a rigid receptor and flexible ligand approach. Glide systematically searches for favorable ligand conformations and orientations within the defined active site through a series of hierarchical filters, including a grid-based search followed by energy minimization and scoring. The final poses were ranked using GlideScore, a proprietary empirical scoring function that approximates ligand-binding free energy and includes energetic terms for van der Waals and electrostatic interactions, hydrogen bonding, and desolvation penalties. The top-scoring pose for each BCAA was selected as the starting conformation for the MD simulations. The two-dimensional (2D) and three-dimensional (3D) ligand interaction diagrams shown in Figure 1 and Figure 2 were generated using the Maestro graphical interface.

### 3.4. Molecular Dynamics (MD) Simulations

All-atom MD simulations were performed using Desmond (version 8.3) (Schrödinger Release 2025-1: Desmond Molecular Dynamics System, D. E. Shaw Research, New York, NY, USA, 2024; Maestro-Desmond Interoperability Tools, Schrödinger, New York, NY, USA, 2025) from the Schrödinger Suite to investigate the structural dynamics and binding mechanisms of SESN2 with its ligands [20].

#### 3.4.1. System Setup

Four distinct systems were prepared for simulation using Maestro (version 14.5) within the Schrödinger Suite (Schrödinger Release 2025-1): apo-SESN2 (unbound), SESN2-Leucine, SESN2-Isoleucine, and SESN2-Valine. Each system was placed in an orthorhombic periodic boundary box and solvated with the TIP3P explicit water model, ensuring a minimum buffer distance of 10 Å between the protein and the box boundaries. The overall charge of each system was neutralized by adding an appropriate number of counter-ions (Na+ or Cl−). The OPLS4 force field was employed for all atoms in the system, as it has demonstrated accuracy in modeling protein–ligand interactions and conformational energetics.

#### 3.4.2. Equilibration Protocol

Prior to production runs, each system underwent a comprehensive, multi-stage relaxation protocol to ensure thermodynamic stability and proper equilibration. The default Desmond protocol involved the following steps: (1) an initial energy minimization using the steepest descent method to remove steric clashes; (2) a short 100 ps simulation using Brownian Dynamics in the NVT ensemble at a low temperature of 10 K, with heavy atoms of the solute (protein and ligand) restrained with a force constant of 50 kcal/mol/Å^2^; (3) a sequence of short simulations (12 ps NVT, 12 ps NPT) at 10 K with continued restraints to introduce temperature and pressure coupling gradually; (4) heating of the system to the target temperature of 310 K over a 12 ps NPT simulation with restraints; (5) a final 24 ps equilibration run in the NPT ensemble with all restraints removed to allow the system to relax fully.

#### 3.4.3. Production Simulations

Following the relaxation protocol, production MD simulations were run for 500 ns each for the four systems. To ensure robust statistical sampling and assess reproducibility, three independent replicates were performed for each system, starting from the same equilibrated structure but with different initial velocities. This resulted in a total simulation time of 1.5 µs for each system. All production simulations were conducted in the NPT ensemble. The temperature was maintained at a constant 310 K using the Langevin thermostat, and the pressure was controlled at 1.01325 bar using the Martyna–Tobias–Klein (MTK) barostat. The RESPA integrator was employed with a time step of 2.0 fs for integrating the equations of motion. Long-range electrostatic interactions were calculated using the Particle Mesh Ewald (PME) method. All simulations were performed on graphics processing units (GPUs), and the system’s coordinates were saved every 100 ps for subsequent analysis.

### 3.5. Trajectory Analysis

Post-simulation analysis was performed on the concatenated trajectories from the three replicates for each system. Trajectory visualization and molecular graphics were generated using the Maestro graphical interface (Schrödinger, LLC, New York, NY, USA, 2025). Analysis was conducted using tools within the Schrödinger Suite (Schrödinger Suite 2025-1) and custom Python scripts (version 3.9). Specifically, these scripts used the MDAnalysis library (version 2.7.0) to read and process trajectory data [21]. The quantitative plots of RMSD, Rg, and RMSF were generated using Matplotlib (version 3.8.0). The DCCM plots were also calculated from the Cα atom coordinates and plotted using these custom Python scripts.

The Cα-RMSF was calculated for each residue to identify regions of high and low flexibility, providing insights into the local dynamic changes upon ligand binding. The Rg was computed over time to measure the overall conformation of the protein, indicating large-scale changes such as expansion or contraction. The interactions between SESN2 and each BCAA, including hydrogen bonds, hydrophobic contacts, and water bridges, were monitored throughout the simulations to identify key residues and quantify the stability of specific contacts. DCCM analysis was performed to quantify the correlated motions between pairs of Cα atoms. The resulting matrices highlight regions of the protein that move together (positively correlated) or in opposition (negatively correlated), revealing pathways of allosteric communication.

### 3.6. Binding Free Energy Calculations (MM/GBSA)

The binding free energies (ΔG_bind_) for the SESN2–BCAA complexes were estimated using the MM/GBSA method as implemented in the Prime module (mmshare version 7.1) of the Schrödinger Suite (Schrödinger Release 2025-1: Prime, Schrödinger, LLC, New York, NY, USA, 2025). This method calculates the binding free energy as the difference between the free energy of the complex and the sum of the free energies of the unbound protein and ligand. MM/GBSA calculations were performed on 100 evenly spaced frames extracted from the final 100 ns of each MD trajectory using the thermal_mmgbsa.py script provided by Schrödinger. The free energy for each species is estimated as the sum of the molecular mechanics energy (E_MM_), a polar solvation term (G_GB_) calculated using the Generalized Born model, and a non-polar solvation term (G_SA_) estimated from the solvent-accessible surface area (SASA). The entropic contribution (−TΔS) was not calculated due to its high computational cost and potential for introducing inaccuracies, a common practice in comparative MM/GBSA studies. The final reported ΔG_bind_ values and their components are averages over the three independent replicates for each system.

### 3.7. Dimensionality Reduction and Free Energy Landscape (FEL) Analysis

The conformational space explored by each system was characterized through Principal Component Analysis (PCA) and density-based clustering. PCA was performed on the Cα atoms of the protein from the concatenated trajectories using Schrödinger’s trj_essential_dynamics.py utility. PCA is a dimensionality reduction technique that identifies the principal modes of collective atomic motion. The analysis was carried out using Schrödinger’s trj_essential_dynamics.py utility with atom selection limited to the receptor and ligand. The trajectories were projected onto the first two principal components (PC1 and PC2), which accounted for the dominant conformational variations.

To provide a quantitative, objective comparison of the conformational landscapes, the projected data for each system were clustered using the Density-Based Spatial Clustering of Applications with Noise (DBSCAN) algorithm, implemented in the scikit-learn library. This method was chosen for its ability to identify arbitrarily shaped clusters and to classify low-density transition-state regions as noise, thereby providing a more physically realistic partitioning of the conformational states. A single, consistent eps parameter of 1.2 and a min_samples value of 4 were used across all four systems to ensure a direct and unbiased comparison. The eps value was determined by analyzing the point of maximum curvature on a k-distance graph (k = 4) generated from the apo-protein trajectory, ensuring that the chosen parameter was appropriate for the most complex landscape.

The Gibbs Free Energy Landscape (FEL) was then constructed using the GROMACS gmx sham module (T = 310 K, 100 energy levels), which estimates the Gibbs free energy ΔG = −kBTln(P), where P is the probability density of each conformation in the PC1–PC2 space. The resulting 2D and 3D landscapes depict stable low-energy conformational states as deep basins and transition states as higher-energy barriers, providing a comprehensive picture of the protein’s conformational dynamics and thermodynamics.

## 4. Conclusions

This comprehensive computational investigation has provided a detailed understanding of molecular mechanisms at multiple levels, from atomic interactions to the collective dynamics of the protein ensemble. Our integrated analysis demonstrates that the high specificity of SESN2 for leucine is not merely a function of static binding affinity. Still, it results from a dynamic process of “conformational locking” that we have quantitatively characterized. The superior shape complementarity of leucine’s isobutyl side chain within the hydrophobic binding pocket maximizes favorable van der Waals energetic contributions. This energetic advantage is sufficient to overcome the entropic penalty of restricting the protein’s intrinsic flexibility, driving a global allosteric transition that stabilizes the entire structure. Our clustering analysis provides definitive evidence for this process, showing that leucine binding reduces the number of accessible conformational states by four-fold and slashes the time spent in transient states by 78%. This signal is propagated through a coordinated network of dynamic correlations, funneling the protein from a flexible, high-entropy apo-ensemble, characterized by a long-tail distribution of 35 microstates, into a singular, stable, low-entropy ensemble dominated by a few well-defined macrostates that represent its signaling-competent form. Crucially, our analysis shows that while other BCAAs, such as valine, can also reduce conformational heterogeneity, they fail to establish the robust allosteric communication network unique to leucine binding and essential for proper signal transduction.

These findings have significant implications beyond the canonical mTORC1 pathway. The identified allosteric network provides a plausible physical channel through which leucine binding can communicate with distal functional sites, potentially modulating SESN2’s mTORC1-independent roles in Nrf2-mediated antioxidant defense and ER stress responses. This offers a potential explanation for the complex, context-dependent biological outcomes of SESN2 activation and may shed light on the recent clinical failure of the direct mTORC1 activator NV-5138. The therapeutic efficacy of SESN2 modulators may not depend solely on mimicking leucine’s affinity but on the ability to recapitulate the specific allosteric state that achieves the correct physiological balance across all of SESN2’s functional outputs.

Looking forward, this work highlights several critical avenues for future research. First, there is an urgent need to experimentally validate the predicted conformational changes. High-resolution cryo-EM studies aimed at capturing the true, flexible apo-state of SESN2—perhaps aided by mutations designed to destabilize the leucine-bound conformation—and the SESN2-GATOR2 complex in both the presence and absence of leucine would be invaluable for confirming the proposed allosteric mechanism. Second, functional studies are required to test the hypothesis that BCAA binding differentially modulates SESN2’s mTORC1-independent functions. Site-directed mutagenesis of key residues within the identified allosteric network, coupled with assays for Nrf2 activation and ER stress markers, could directly probe the functional connectivity of these pathways. Finally, our structural and dynamic model provides a powerful platform for next-generation therapeutic design. Future efforts should move beyond simple orthosteric mimetics to develop allosteric modulators. Such compounds could be designed to selectively stabilize specific conformations of SESN2, thereby fine-tuning its functional outputs to achieve a desired therapeutic effect—for example, maximizing its antioxidant and anti-inflammatory roles while minimizing mTORC1 activation in the context of neurodegenerative disease. This approach holds the promise of developing more refined and effective therapies for the vast array of diseases in which SESN2 plays a pivotal role.

## Figures and Tables

**Table 1:** MM/GBSA Binding Free Energy and Component Analysis for BCAA-SESN2 Complexes. All energy values are in kcal/mol and represent the average ± standard deviation from three independent 500 ns MD simulations. ΔG_bind_ is the total binding free energy. ΔEvdW and ΔE_Coulomb_ are the van der Waals and electrostatic contributions, respectively. ΔG_Lipo_ and ΔG_SolvGB_ are the non-polar and polar solvation free energy terms. E_StrainLig_ is the strain energy of the ligand in its bound conformation.

Energy Term (kcal/mol)	Leucine	Isoleucine	Valine
ΔG_bind_ (MMGBSA)	−37.60 ± 2.39 *	−34.47 ± 1.98	−30.32 ± 3.18
ΔE_vdW_	−28.09 ± 1.28	−25.92 ± 1.38	−21.96 ± 1.29
ΔE_Coulomb_	13.13 ± 3.33	14.59 ± 2.89	11.27 ± 2.15
ΔG_Lipo_	−8.25 ± 0.59	−7.42 ± 0.45	−5.92 ± 0.61
ΔG_SolvGB_	−12.30 ± 1.87	−13.12 ± 1.55	−11.10 ± 1.93
E_StrainLig_	0.66 ± 0.62	0.52 ± 0.27	0.41 ± 0.32

* The difference in ΔG_bind_ between Leucine and Valine is statistically significant (*p* < 0.05) based on an independent two-sample *t*-test.

**Table 2:** Comparative Analysis of Conformational Landscapes via DBSCAN Clustering. Quantitative results from the density-based clustering of the conformational space sampled by each system. “# Stable States” is the number of distinct conformational clusters identified. “% Noise (Transitions)” is the percentage of simulation time spent in low-density regions between stable states. “% in Largest State” is the population of the single most-populated conformational state. The data quantify the dramatic reduction in conformational heterogeneity upon binding of leucine and valine.

System	# Stable States	% Noise (Transitions)	% in Largest State
Apo Form	35	11.6%	36.1%
Isoleucine-Bound	27	11.2%	33.6%
Valine-Bound	9	4.2%	33.6%
Leucine-Bound	9	2.6%	33.5%

## Data Availability

The datasets generated and analyzed during the current study, including all simulation trajectories and analysis files, are openly available in the Zenodo repository at https://doi.org/10.5281/zenodo.16835187 (accessed on 1 December 2025).

## Abbreviations

The following abbreviations are used in this manuscript:

AMPK	AMP-activated Protein Kinase
BCAA	Branched-Chain Amino Acid
Cryo-EM	Cryogenic Electron Microscopy
DCCM	Dynamic Cross-Correlation Matrix
ER	Endoplasmic Reticulum
FEL	Free Energy Landscape
GAP	GTPase-Activating Protein
GATOR	GAP Activity Towards Rags
Kd	Dissociation Constant
MD	Molecular Dynamics
MM/GBSA	Molecular Mechanics/Generalized Born Surface Area
mTORC1	Mechanistic Target of Rapamycin Complex 1
mTORC2	Mechanistic Target of Rapamycin Complex 2
NRF2	Nuclear Factor Erythroid 2-related Factor 2
PCA	Principal Component Analysis
PI3K	Phosphatidylinositol 3-Kinase
Rg	Radius of Gyration
RMSD	Root-Mean-Square Deviation
RMSF	Root-Mean-Square Fluctuation
SESN2	Sestrin2
